# Linking the multiple-demand cognitive control system to human electrophysiological activity

**DOI:** 10.1016/j.neuropsychologia.2025.109096

**Published:** 2025-04-15

**Authors:** Runhao Lu

**Affiliations:** MRC Cognition and Brain Sciences Unit, University of Cambridge, United Kingdom

**Keywords:** Multiple-demand network, Cognitive control, Neural oscillations, Aperiodic neural activity, Functional connectivity

## Abstract

The frontoparietal multiple-demand (MD) network serves as a core system for domain-general cognitive control, with robust activation with increased demand across diverse tasks. While fMRI studies have characterised the MD network's role in cognitive demand, linking these findings to electrophysiological activity remains a critical challenge. This article discusses the potential of oscillatory and aperiodic neural activity to bridge this gap. Although recent meta-analyses highlight mid-frontal theta power as a robust marker of task demand, its localised spatial distribution, limited cross-task generalisability, and potential confounds from aperiodic components limit its ability to fully represent the MD network. In contrast, aperiodic activity, particularly broadband power, has emerged as a strong candidate for indexing task demand due to its robust decoding performance and cross-task generalisability in response to diverse task demands, and spatial overlap with MD regions. Aperiodic activity may reflect fundamental neural properties, such as spiking rates and excitation/inhibition (E/I) balance, and is scale-free and exists across modalities, positioning it as a promising mechanism underpinning domain-general cognitive control that links to the MD network. Meanwhile, multiplexed low-frequency oscillations (e.g., delta and theta) may implement inter-regional synchronisation within the MD network, enabling large-scale coordination between MD subregions that supports cognitive control. Together, this article proposes a hypothetical framework linking the MD network to electrophysiological responses: Aperiodic broadband power, potentially reflecting population-level spiking activity, may support activation within MD regions, while multiplexed low-frequency oscillatory synchronisations may mediate inter-regional connectivity between MD regions.

## Highlights

• The MD network serves as the core system for domain-general cognitive control.
• Linking oscillatory power to the MD network remains significant challenges.
• Aperiodic broadband power is a strong candidate underpinning the MD network.
• Increased MD activation might align with a decrease in broadband power.
• Low-frequency oscillations may coordinate connectivity between MD regions.

## 1 Introduction

Human fMRI studies have consistently suggested the multiple-demand (MD) network as a central system for domain-general cognitive control, referring to the brain's ability to regulate and coordinate goal-directed behaviour in response to varying cognitive demands (Duncan, 2010; Duncan et al., 2020). This network, also commonly referred to as the frontoparietal control network or the task-positive network, spans distributed but highly specific regions in the frontal, parietal, and temporal cortices (Assem et al., 2020; Fedorenko et al., 2013). MD network activation is robustly associated with cognitive demands across diverse tasks and exhibits an adaptive coding property, allowing flexible representation of task-relevant information to meet varying cognitive challenges (Duncan, 2001; Duncan et al., 2020; Woolgar et al., 2011; Woolgar et al., 2016).

In parallel, numerous studies using MEG or EEG have explored electrophysiological responses, particularly event-related oscillatory activity (Makeig et al., 2004), underlying cognitive control across different tasks. These event-related oscillations are analysed in response to discrete task events (e.g., stimulus onset, cognitive demand manipulation), reflecting transient neural dynamics associated with cognitive control processes. Evidence indicates that oscillatory dynamics across a broad range of frequency bands, from delta (∼0.5–3 Hz) to gamma (>30 Hz), may exhibit demand-related effects, with power either increasing or decreasing in more demanding conditions (Chikhi et al., 2022; Pavlov and Kotchoubey, 2022). However, despite extensive research examining fMRI and MEG/EEG correlates of task demands, significant challenges remain in linking the robust MD activations observed in fMRI studies to these electrophysiological findings.

**Fig. 1:**
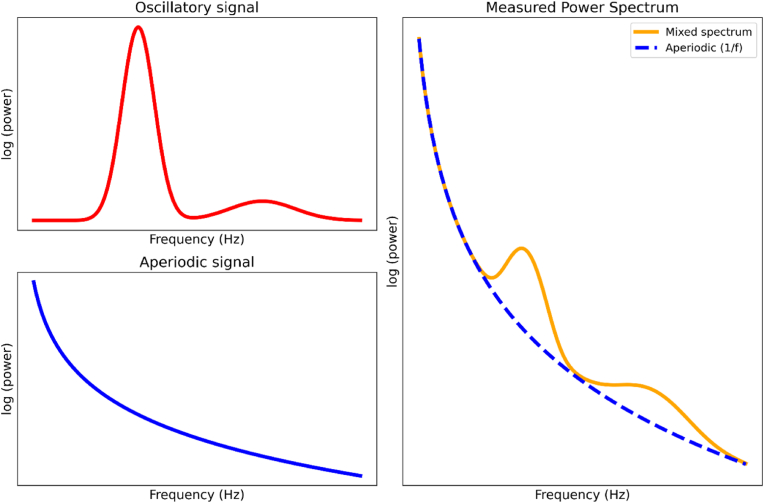
Decomposition of the MEG/EEG power spectrum into oscillatory and aperiodic components. The left panels illustrate the pure oscillatory (top) and aperiodic signal (bottom). The right panel shows the measured power spectrum (orange) as a mixture of oscillatory and aperiodic components. The aperiodic signal (blue dashed line) reflects the 1/*f* background, while the mixed spectrum (orange) incorporates oscillatory peaks with the aperiodic background.

This article first reviews recent meta-analyses studies on the relationship between oscillatory power and task demands, which suggest frontal theta power (∼4–8 Hz) as the strongest oscillatory candidate for supporting domain-general cognitive control. Nevertheless, substantial challenges persist in linking frontal theta to MD activity due to its spatial localisation and potential confounds from other neural components. This article further proposes that aperiodic neural activity, another key component of the MEG/EEG power spectrum (Fig. 1), may reflect fundamental neural mechanisms, such as spiking rates and excitation/inhibition (E/I) balance, offering a more compelling electrophysiological basis for MD network activation. As strong inter-regional connectivity is another key feature of the MD network (Assem et al., 2020), I further discuss its potential electrophysiological mechanisms, focusing on the role of long-range low-frequency (e.g., delta and theta) synchronisations in coordinating activity between distributed MD regions. Together, with consideration of both aperiodic activity and long-range functional connectivity (FC), it may shed light on the electrophysiological underpinnings of the MD network and offer a potential framework for understanding how local and global neural dynamics support domain-general cognitive control.

## 2 Linking oscillatory power to MD activations

### 2.1 Frontal theta: a strong candidate for domain-general cognitive control

Much like the robust activation of the MD network in response to task demand across diverse contexts, there has been significant interest in the MEG/EEG literature in identifying reliable neural metrics supporting cognitive control in various tasks. Among these, the spectral power of neural oscillations across different frequency bands has been a prominent focus (Cavanagh and Frank, 2014; Chikhi et al., 2022; Pavlov and Kotchoubey, 2022; Roux and Uhlhaas, 2014). However, despite extensive investigation, findings in this area have often been inconsistent and sometimes challenging to interpret.

A recent meta-analysis by Chikhi et al. (2022) reviewed 45 effect sizes from 24 studies involving 723 participants to examine the relationship between oscillatory power and cognitive demand across diverse tasks. The results indicated that increased cognitive demand is associated with increases in theta power (*g* = 0.68, CI [0.41, 0.95]), decreases in alpha (∼8–12 Hz) power (*g* = −0.25, CI [−0.45, 0.04]), and increases in beta (∼15–30 Hz) power (*g* = 0.50, CI [0.21, 0.79]). Among these, theta power demonstrated the highest effect size and emerged as the most robust and reliable index for tracking cognitive demand (Cavanagh and Frank, 2014; Cohen and Donner, 2013; Cooper et al., 2017; Jensen and Tesche, 2002; Ratcliffe et al., 2022). Furthermore, increased theta power during cognitive tasks is associated with improved task performance (Tan et al., 2024), which emphasises its significant role in cognitive processes directly linked to behaviour.

Similarly, Pavlov and Kotchoubey (2022) conducted a meta-analysis of 100 MEG/EEG studies exploring the relationship between oscillatory power and working memory load, which specifically refers to the amount of information temporarily held and processed in memory, with higher loads imposing greater cognitive demands. Their findings reinforced theta power increases as the most consistent neural marker of cognitive demand including working memory load.

In contrast, according to both meta-analyses (Chikhi et al., 2022; Pavlov and Kotchoubey, 2022), while cognitive demand (including working memory load) typically influences alpha power as well, its direction is often inconsistent. Some studies report increases in alpha power with demand (Jensen, 2024; Jensen et al., 2002; Klimesch, 1999), while others observe decreases (Brouwer et al., 2014; Grissmann et al., 2017). These discrepancies may be driven by multiple factors such as stimulus presentation style (simultaneous vs. successive) (Hsieh et al., 2011), task content (verbal vs. visual) (van Ede, 2018), or differing neural sources of alpha activity (Rodriguez-Larios et al., 2022). As a result, alpha power appears less generalisable across different tasks in tracking cognitive demand compared to the robust MD activation observed in fMRI studies across a wide range of tasks (Assem et al., 2022; Fedorenko et al., 2013). Beta power, while moderately associated with cognitive demand as suggested by meta-analyses, exhibits significant variability in spatial and frequency distributions (Altamura et al., 2010; Orlandi and Brooks, 2018; Pavlov and Kotchoubey, 2017), reducing its reliability as a general neural marker of cognitive control. High-frequency gamma power, which reflects local spiking rates (Vinck et al., 2024; Voytek and Knight, 2015), is another potential candidate. However, due to the low signal-to-noise ratio of non-invasive MEG/EEG recordings, gamma activity has been less studied, with existing studies reporting inconsistent results (Brookes et al., 2011; Poch et al., 2014).

### 2.2 Challenges in linking frontal theta power to the distributed MD network

Although frontal theta power demonstrates a strong association with diverse cognitive demands, significant challenges remain in linking it to the MD network. The most prominent issue lies in the differences between their cortical patterns. MEG/EEG source localisation and simultaneous EEG-fMRI studies consistently indicate that demand-related frontal theta power originates primarily from medial frontal regions, such as the anterior/mid-cingulate cortex (ACC/MCC) and the pre-supplementary motor area (pre-SMA) (Brookes et al., 2011; Meltzer et al., 2007; Michels et al., 2010; Onton et al., 2005). While the MD network includes a medial frontal patch encompassing these areas, it extends to eight functionally similar patches distributed across the lateral frontal, parietal, and temporal cortices (Assem et al., 2020; Fedorenko et al., 2013). Notably, intracranial EEG studies have found that theta power in medial frontal areas (e.g., dorsal ACC) and dorsolateral prefrontal cortices (DLPFC) can exhibit opposite patterns with memory load (Brzezicka et al., 2019), suggesting potential distinct functional roles of theta oscillations across MD regions. Thus, mid-frontal theta power may not account for the electrophysiological correlates underpinning the entire MD network. Instead, it might reflect the activity of a medial frontal subsystem within the broader MD network, while oscillations in other regions, such as parietal alpha or lateral frontal theta, might underpin additional MD subsystems. Supporting this view, recent studies suggested that frontal theta and posterior alpha work together to support cognitive control, with the frontal theta associated with target prioritisation and posterior alpha linked to distractor suppression (Riddle et al., 2024; Riddle, Scimeca, Cellier, Dhanani, & D'Esposito, 2020).

**Fig. 2:**
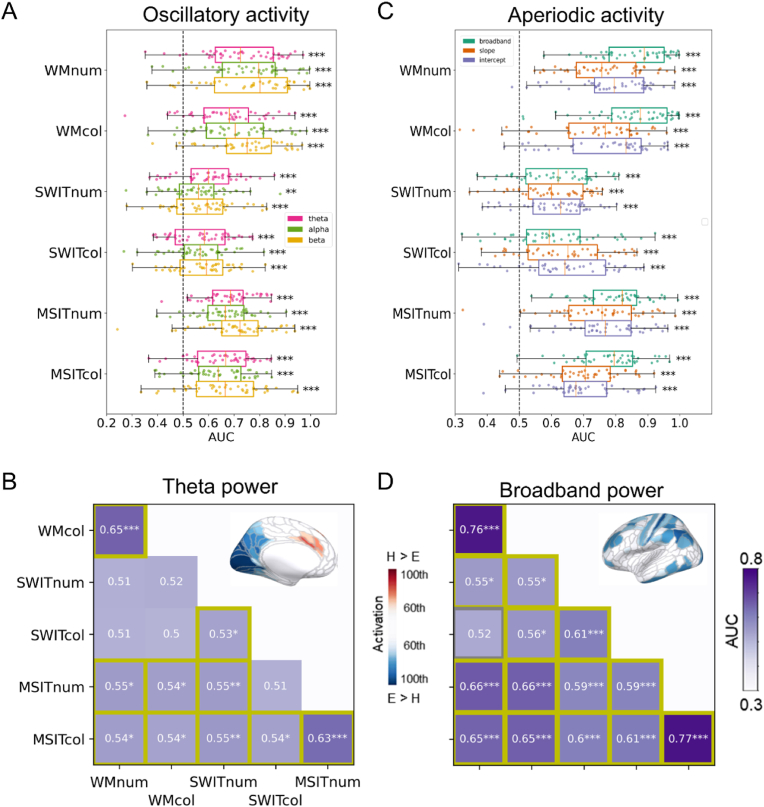
Decoding task demand (hard vs. easy) and cross-task generalisability for oscillatory and aperiodic activity (Lu et al., 2024). (A) Decoding performance (area under the curve, AUC) of task demand (hard vs. easy) using oscillatory power (theta, alpha, and beta) across six subtasks. Each dot means one participant. Error bars represent standard errors. (B) Cross-task generalisation of task demand based on theta power. Yellow borders highlight significantly above-chance AUC values, indicating generalisability across the respective subtasks. The inset brain image shows the averaged source patterns, revealing mid-frontal theta power increases during hard task conditions. (C) Decoding performance of task demand using aperiodic components (broadband power, slope, and intercept) for each subtask. (D) Cross-task generalisation of task demand based on aperiodic broadband power. The inset brain image shows the averaged source patterns, illustrating a widespread frontoparietal broadband power decrease during hard task conditions. WM: Working memory task; SWIT: Switching task; MSIT: Multi-source interference task; num: Alphanumeric task; col: Colour task. ∗*p* < 0.05, ∗∗*p* < 0.01, ∗∗∗*p* < 0.001 (FDR-corrected).

Beyond localisation differences, another challenge arises from the potential confound of aperiodic neural activity. Recent evidence suggests that modulations traditionally attributed to theta oscillations may, in fact, reflect changes in aperiodic neural activity. For instance, a recent study disentangled oscillatory and aperiodic effects during a working memory task (Frelih et al., 2024). Surprisingly, they found that a substantial proportion of the modulations previously attributed to frontal theta oscillations were better explained by changes in aperiodic activity. Similarly, our recent MEG/EEG study (Lu et al., 2024) used multivariate pattern analysis (MVPA) to investigate the roles of oscillatory power and aperiodic activity in encoding task demand across six subtasks, including working memory, task switching, and multi-source interference tasks presented in either alphanumeric or colour formats. This study also assessed the cross-task generalisability of these neural signals. If a neural response supports domain-general cognitive control, as the MD network does, it should exhibit a generalisable pattern in classifying task difficulty across different contexts. While results suggested that oscillatory theta power, along with alpha and beta power, reliably represented demand information within individual subtasks (Fig. 2A), these patterns did not generalise across all subtasks (Fig. 2B), raising questions about their role in domain-general cognition.

In summary, although frontal theta power emerges as the most robust metric for tracking task demand across many cognitive control processes, linking it to the MD network presents several challenges, including differences in source distribution, the potential confounds of aperiodic components, and limited cross-task generalisability.

## 3 Linking aperiodic neural activity to MD activations

### 3.1 Estimation and physiological interpretation of aperiodic neural activity

MEG/EEG power spectra consist of a combination of components, including a broadband 1/*f*-like aperiodic signal and narrow-band oscillations (Fig. 1) (Buzsáki et al., 2012; He, 2014; Voytek and Knight, 2015). Separating these components is crucial for understanding their roles in cognition, as traditional measures of narrow-band oscillatory power can be significantly confounded by broadband shifts or spectral rotations caused by aperiodic activity (Donoghue et al., 2020).

A key feature of aperiodic activity is that, when plotted in log-log space, it approximates a straight line, allowing it to be modelled with a simple linear function to extract its slope and intercept. The slope of this line is equivalent to the negative aperiodic exponent (x), which describes the rate of spectral decay in the form of 1/*f* ^x^. The intercept, on the other hand, represents the overall power offset. Several established methods exist to disentangle aperiodic and oscillatory activity from the mixed signal (Donoghue et al., 2024), including parametric approaches such as Fitting Oscillations & One Over F (FOOOF) (Donoghue et al., 2020) and non-parametric methods like irregular resampling auto-spectral analysis (IRASA) (Wen and Liu, 2016). These techniques estimate aperiodic activity by quantifying its slope and intercept on a log-log scale. In addition, they can compute aperiodic broadband power as the sum or average power across a broad frequency range, typically derived from the raw aperiodic spectrum that is not log-log transformed. Both approaches yield robust and comparable estimations in general, though they each have their advantages and limitations (Cross et al., 2022; Gerster et al., 2022). It should be noted that, while the aperiodic intercept and broadband power are theoretically highly related, their exact relationship depends on the method used to estimate aperiodic activity. When using models that fit aperiodic activity in log-log space, the intercept directly represents broadband power. However, when broadband power is calculated from the raw aperiodic spectrum (e.g., after IRASA) without enforcing a linear function, it may be influenced by both intercept and slope, capturing additional variability in neural activity.

Aperiodic activity is ubiquitous across a wide range of modalities, including local field potentials (LFP), MEG/EEG, and fMRI blood oxygen level dependent (BOLD) signals including resting-state network dynamics (Bullmore et al., 2001; Fransson et al., 2013; He, 2011, 2014; Maxim et al., 2005). This cross-modality presence may offer a significant advantage that allows for comparability between electrophysiological and imaging studies.

Despite its ubiquity, interpreting the mechanisms underlying 1/*f*-like aperiodic activity remains challenging. One of the most widely accepted interpretations links the aperiodic slope to the synaptic E/I ratio (Gao et al., 2017; Trakoshis et al., 2020). A steeper slope reflects increased inhibition (lower E/I ratio), while a flatter slope indicates increased excitation (higher E/I ratio) (Gao et al., 2017). This interpretation holds across modalities, including both electrophysiological and fMRI signals (Chini et al., 2022; Trakoshis et al., 2020). Alternative explanations for aperiodic slope include neural complexity (Mostile et al., 2019), criticality in brain networks (Kardan et al., 2020; Palva et al., 2013), and neural noise (Dave et al., 2018), though these are relatively less studied compared to the E/I ratio hypothesis (Donoghue, 2024).

For aperiodic broadband power, which correlates closely with the intercept, evidence strongly suggests a positive relationship between high-frequency broadband power (e.g., >70 Hz) and neuronal spiking rates (Fries et al., 2001; Pesaran et al., 2002; Voytek and Knight, 2015). Additionally, an intracranial recording study in human patients extended this relationship across a broader frequency range (2–150 Hz), encompassing both low and high frequencies (Manning et al., 2009).

Taken together, aperiodic activity provides crucial information about fundamental neural properties such as population-level spiking rates (via broadband power/intercept) and E/I balance (via slope) (Vinck et al., 2024). Combined with its scale-free nature across modalities, aperiodic neural activity stands out as a strong candidate for underpinning diverse cognitive processes across both electrophysiological responses and BOLD signals.

### 3.2 Functional roles of aperiodic activity in cognitive control

Although the aperiodic 1/*f*-like background is historically considered as noise, an increasing number of studies highlight its importance in supporting diverse cognitive processes and behaviours (Donoghue et al., 2020; He, 2014). For example, aperiodic activity has been found to correlate with ageing (Dave et al., 2018; Donoghue et al., 2020; Kałamała et al., 2024; Voytek et al., 2015), sleep stages (Lendner et al., 2020), levels of consciousness (Colombo et al., 2019), psychiatric diseases such as ADHD (Robertson et al., 2019) and schizophrenia (Molina et al., 2020), and various aspects of cognitive performance (Canales-Johnson et al., 2021; Churchill et al., 2016; Gyurkovics et al., 2022; He, 2014; Kardan et al., 2020; Ouyang et al., 2020). Notably, these findings extend across both resting-state and task-processing conditions, underscoring the relevance of aperiodic activity to cognition and behaviour in different states.

Within task-processing states, aperiodic activity has been investigated in relation to both sustained (tonic) cognitive states and transient (event-related) responses, with increasing evidence suggesting that it reliably tracks task demand across different cognitive tasks. Pei et al. (2023) examined demand-related changes in a sustained mental arithmetic task, where participants silently counted backward for 60 s under two conditions: an easy task, in which they subtracted 1 from 100, and a hard task, in which they subtracted 7 from 300. Aperiodic features measured over the full duration outperformed oscillatory metrics (e.g., event-related oscillatory power and phase-amplitude coupling) in tracking cognitive demand, with more demanding conditions exhibiting a reduction in the intercept and a flatter slope. In contrast, several studies have investigated event-related aperiodic modulation in response to transient task demands. For example, Kardan et al. (2020) examined scale-invariance during working memory retention (∼1 s) and found that the Hurst exponent (*H*; an indicator of the scale-free aperiodic property) decreased with increasing working memory load, indicating a flattening of the 1/*f* slope. Similarly, fMRI studies have demonstrated that BOLD signal dynamics become less scale-free (decreased *H*) with increased cognitive effort (Churchill et al., 2016; He, 2011). These findings align with a theoretical framework suggesting that the brain, when at rest, operates near a critical state, which optimises information storage and transfer. During demanding tasks, the brain departs from this criticality, suppressing scale-free dynamics to allocate resources to task-specific processes. In addition, Donoghue et al. (2020) applied the FOOOF method to EEG signals recorded during working memory maintenance period (around 0.5 s). They found an event-related decrease of intercept (reflecting a downward shift in broadband power) and a flattening of the aperiodic slope compared to baseline. Interestingly, in other types of cognitive control tasks, such as task switching and inhibitory control, studies have reported event-related decreases in broadband power but steeper slopes under higher task demands (Jia et al., 2024; Yan et al., 2024), suggesting task-specific modulations of aperiodic components.

In summary, across cognitive control tasks, harder conditions are generally associated with decreased broadband power (or intercept). The aperiodic slope, however, may appear to vary with task type—flattening in working memory tasks but steepening in task-switching or inhibitory control tasks. Importantly, these modulations occur at both tonic (sustained) and event-related (transient) timescales, suggesting that aperiodic activity tracks task demand dynamically across different cognitive states. These findings highlight the functional significance of aperiodic activity in tracking demand-related cognitive control, with broadband power decrease emerging as a stable index of task effort.

### 3.3 Aperiodic broadband power as a compelling candidate underpinning the MD network

Our recent MEG/EEG study (Lu et al., 2024) systematically examined the contributions of event-related aperiodic and oscillatory activity in domain-general cognitive control across six subtasks, including working memory, task switching, and multi-source interference tasks, each presented in either alphanumeric or colour formats with varying levels of cognitive demand. Using IRASA to disentangle aperiodic (3–30 Hz broadband power, slope, and intercept) and oscillatory [theta (3–7 Hz), alpha (8–12 Hz), and beta (15–30 Hz)] components, we analysed data within a 1.2 s window following stimulus onset, with phase-locked event-related potentials subtracted. We then applied MVPA to decode task demand (hard vs. easy) for each subtask. Results showed that while all oscillatory and aperiodic components contributed to demand encoding (Fig. 2A and C), aperiodic broadband power exhibited the highest decoding performance and the strongest cross-task generalisability in representing task demands (Fig. 2D). This suggests that broadband power calculated from raw aperiodic spectrum is a robust marker of task demand, likely because it potentially captures both intercept and slope dynamics rather than being constrained to a single parameter. Source patterns of broadband power showed a frontoparietal distribution, partially overlapping with the MD network.

To address potential spatial resolution limitations in MEG/EEG, we conducted a follow-up fMRI study (Lu, Assem, Duncan, Woolgar, unpublished) using the same task set. By combining MEG and fMRI data with a model-based fusion approach (Hebart et al., 2018; Moerel et al., 2024), we found that aperiodic broadband power demonstrated the greatest commonality with task demand in MD regions, outperforming oscillatory power and aperiodic slope. These findings suggest that aperiodic broadband power aligns closely with the core characteristics of the MD network, including responding to task demand across diverse tasks, cross-task generalisable coding patterns, and a widespread frontoparietal cortical distribution.

Notably, while fMRI studies typically report increased MD activation with higher task demand, aperiodic broadband power or intercept appears to decrease (Donoghue et al., 2020; Lu et al., 2024; Pei et al., 2023). Consistently, a recent study using simultaneous EEG-fMRI during resting state found that aperiodic EEG components were negatively correlated with BOLD activation in frontal and parietal regions (Jacob et al., 2021). To interpret this directional difference in how broadband power and fMRI BOLD signals respond to task demand, it is helpful to consider their relationship with neuronal spiking rates. Spiking activity has been proposed to positively correlate with broadband power (Manning et al., 2009). However, evidence directly linking task demand to neuronal spiking rates in MD regions is limited. One recent study recorded human single-neuron activity during a working memory task and found that neurons in the pre-SMA, an MD region, were most active during low-demand conditions compared to higher demand conditions (Kaminski et al., 2017). This suggests that robust MD activation in response to increasing task demands might not necessarily equate to increased firing rates within these regions. In other words, fMRI BOLD responses and spiking rates (and consequently broadband power) may exhibit opposing trends during cognitive tasks.

As a more accessible proxy for population-level spiking rates, high-frequency gamma power and transient gamma bursts provide additional insights into this relationship (Carver et al., 2019; Lundqvist et al., 2018; Lundqvist et al., 2016; Pesaran et al., 2002). Recent EEG studies have shown that gamma bursts increase during working memory encoding and decrease during the maintenance delay period, while alpha and beta bursts exhibit the opposite pattern (Kavanaugh et al., 2024). These findings align with results from nonhuman primate studies (Lundqvist et al., 2016) and suggest that population-level spiking rates, indexed by gamma bursts, may decrease during memory maintenance—a period during which fMRI BOLD signals within MD regions typically show increased activity (Li, O'Sullivan and Mattingley, 2022). Supporting this interpretation, an intracranial EEG study found decreased broadband power in the DLPFC, a key MD region, during memory maintenance compared to baseline (Brzezicka et al., 2019). These findings support the idea that MD activation does not necessarily correspond to increased spiking rates or broadband power (though this may occur in certain contexts, e.g., Assem et al. (2023)). This potential distinction is reasonable, as electrophysiological and BOLD signals, while both capturing aspects of domain-general systems, may reflect different underlying processes: BOLD signals are thought to reflect metabolic demands and regional activations, whereas aperiodic electrophysiological signals likely index population-level firing rates and E/I balance.

Taken together, aperiodic neural activity, particularly broadband power, emerges as a compelling candidate for understanding the electrophysiological basis of the MD network and its role in domain-general cognitive control. By potentially reflecting fundamental neural properties such as population-level spiking rates and E/I balance, aperiodic activity provides a scale-free, cross-modality metric for studying brain function. Evidence from MEG/EEG and fMRI studies highlights its ability to track task demands and cognitive effort across diverse contexts, with aperiodic broadband power showing particularly strong associations with demand-related processes and spatially overlapping with MD regions. These characteristics position aperiodic broadband activity as a strong candidate underpinning the MD network.

## 4 Synchronisation between MD regions in low-frequency oscillations

Beyond robust activation within the MD network during demanding tasks, another key feature of the MD network is its strong FC across its regions (Assem et al., 2020). In electrophysiological studies using MEG or EEG, FC is often derived from phase synchrony within specific frequency bands or through cross-frequency coupling, reflecting oscillation-based connectivity (Bastos and Schoffelen, 2016). Alternatively, FC can also be characterised by non-oscillatory activity using information-theoretic methods like weighted symbolic mutual information (wSMI) (King et al., 2013; Vinck et al., 2023). Both oscillatory and non-oscillatory FC may provide complementary information about inter-regional network interactions, offering a more comprehensive understanding of the functional organisation of large-scale brain networks (Imperatori et al., 2019; Vinck et al., 2023).

Although challenges persist in directly linking oscillatory power to the MD network, oscillatory activity may still play a critical role in supporting large-scale connectivity between MD regions. Neural oscillations are thought to enable the coordination of activity between distributed brain regions, forming the basis of large-scale network communication (Buzsáki et al., 2012; Buzsáki and Draguhn, 2004). Specifically, low-frequency oscillations, such as delta and theta bands, are proposed to mediate synchronisation between network subregions and support cognitive control (Helfrich et al., 2019; Helfrich and Knight, 2016).

Many studies have demonstrated that inter-regional theta synchronisation between MD-like regions, such as frontal and parietal cortices or lateral and medial frontal areas, increases with cognitive demand and supports top-down cognitive control (Adamczyk and Wyczesany, 2023; Cavanagh et al., 2009; Cooper et al., 2015; Mizuhara and Yamaguchi, 2007; Oehrn et al., 2014; Polania et al., 2012; Sauseng et al., 2010; Sauseng et al., 2006). Recent work has refined our understanding of how different low-frequency oscillations mediate connectivity between MD-like subregions. For example, Pagnotta, Riddle, and D'Esposito (2024) combined EEG and fMRI data to investigate oscillatory dynamics underlying hierarchical cognitive control. They found that delta band connectivity between lateral frontal and parietal areas supports the control of rule abstraction, whereas theta band connectivity between mid-frontal and parietal regions supports the control of stimulus-action associations. These findings suggest that multiplexed low-frequency synchronisations may allow distinct frontoparietal circuits within the MD network to coordinate diverse aspects of cognitive control. As a follow-up, Riddle et al. (2024) provided causal evidence by combining transcranial magnetic stimulation (TMS) with EEG. They found that theta-frequency TMS to prefrontal cortex increased theta band FC in the frontoparietal network and improved working memory capacity, highlighting the role of theta synchronisation in prioritising memory representations.

In contrast to oscillatory-based connectivity, non-oscillatory FC has received less attention but holds considerable promise. For example, studies have shown that non-oscillatory FC metrics such as wSMI outperform oscillatory-based FC in distinguishing perceptual interpretations of bistable stimuli (Canales-Johnson et al., 2020) and differentiating states of consciousness (King et al., 2013; Sitt et al., 2014). Despite these advances, the role of non-oscillatory FC in the MD network remains underexplored. Investigating whether non-oscillatory FC contributes uniquely to MD connectivity, particularly in the coordination of task demands across distributed regions, is an important avenue for future research. Furthermore, integrating oscillatory- and non-oscillatory-based FC approaches may offer a more comprehensive framework for understanding the functional architecture of the MD network.

In summary, low-frequency oscillatory synchronisations, particularly in the delta and theta bands, likely play a key role in coordinating MD subregions and enabling inter-regional connectivity to support cognitive control. Connectivity between different MD regions might depend on distinct frequency bands (Pagnotta et al., 2024) or involve the same frequency bands but with different functions across regions (Brzezicka et al., 2019). Additionally, non-oscillatory connectivity may also contribute to coordination between MD regions, but further investigations are needed.

## 5 Conclusions and future directions

In this article, I discuss the challenges and possibilities of linking human electrophysiological signals to the MD network. While neural metrics reflecting demand-relayed cognitive control have been extensively studied in both fMRI and MEG/EEG literature, the precise relationships between these measures remain inconclusive. Based on recent meta-analyses, mid-frontal theta power appears to be a robust and reliable feature associated with task demand across multiple contexts, surpassing other frequency bands. However, frontal theta power alone may not account for the entire MD network due to its spatial localisation and limited cross-task generalisability. In contrast, aperiodic activity, particularly broadband power, has emerged as a more promising candidate for indexing task demand. Its robust decoding performance across diverse tasks, spatial overlap with MD regions, and strong cross-task generalisability position it as a reliable neural metric. Aperiodic activity likely reflects fundamental neural mechanisms, such as population-level spiking rates and E/I balance, and its scale-free nature across modalities (e.g., LFPs, MEG/EEG, and fMRI) makes it well-suited for underpinning diverse cognitive processes and comparable across imaging methods. At the network level, multiplexed low-frequency oscillations (e.g., delta and theta) likely play a critical role in synchronising distributed MD regions, enabling large-scale coordination of cognitive functions.

This article proposes a possible framework for the electrophysiological mechanisms underlying the MD network. At the local level, MD activations in response to task demand may be associated with decreased aperiodic broadband power and, potentially, reduced spiking rates of specific neuronal populations within the same area. These aperiodic modulations occur across both tonic (sustained) and event-related (transient) timescales, reflecting dynamic neural adaptations to cognitive effort. At the network level, synchronisation in low-frequency oscillations may coordinate communication within the MD network, with different frequencies (or the same frequency serving distinct functions) mediating connectivity across different MD regions to facilitate large-scale integration of cognitive control processes. Thus, aperiodic and oscillatory activity may work together in supporting domain-general cognitive control, with aperiodic activity reflecting local neural excitability and resource allocation, while low-frequency oscillations facilitate large-scale communication across MD regions.

One potential limitation of this framework is the selected frequency range (<30 Hz) used to extract aperiodic components in many studies (e.g., Lu et al., 2024). This range was chosen for its robust detectability in non-invasive MEG/EEG and its alignment with low-frequency oscillatory dynamics linked to cognitive control. However, it does not capture higher frequencies (>30 Hz), which may contain additional information about E/I balance and local spiking activity and provide a better estimation of aperiodic slope. Future research, particularly using intracranial recordings, should examine aperiodic features across a broader frequency range and compare how aperiodic activity differs between lower (3–30 Hz) and higher (>30 Hz) frequency bands. Such studies could further clarify the role of aperiodic dynamics across different neural scales and refine our understanding of how aperiodic activity supports MD network function in domain-general cognitive control.

To empirically test or falsify this framework, future research should implement targeted experimental approaches that examine the relationship between aperiodic features, oscillatory synchronisation, and MD network function. Table 1 outlines key research questions, methodological approaches, and testable hypotheses that can help evaluate the proposed electrophysiological mechanisms underlying the MD network.

**Table 1:** Experimental approaches to test and falsify the proposed framework.

Research question	Methodology	Hypotheses
Is aperiodic broadband power a domain-general marker of cognitive demand?	Use electrophysiological methods with MVPA and cross-task generalisation across more diverse cognitive tasks.	If aperiodic broadband power is a domain-general marker, it should predict cognitive demand consistently across a more diverse range of cognitive tasks. Otherwise, it may suggest a more task-specific role rather than domain-general.
Does broadband power in the 3–30 Hz range reflect a genuine broadband effect, or is it influenced by the slope flattening across a broader frequency range?	Use intracranial EEG covering both low (<30 Hz) and high (>30 Hz) frequency bands to determine whether broadband power remains a robust index when accounting for the broader spectrum.	If broadband power genuinely reflects domain-general cognition, its effects should persist even when higher frequency bands are included in the analysis. If broadband power effects disappear while aperiodic slope effects become stronger with higher frequencies included, this would suggest that the observed effects are due to slope flattening rather than true broadband power changes.
Does aperiodic broadband power in the low frequency range (3–30 Hz) correlate with spiking activity in MD regions?	Perform intracranial EEG and single-unit recordings in human patients or non-human primates performing cognitive tasks to test direct correlations between broadband power and spiking rates.	If aperiodic broadband power (3–30 Hz) reflects population-level firing rates, it should positively correlate with local spiking rates and high-frequency gamma power in intracranial recordings. If no significant correlation is found between aperiodic broadband power and spiking activity, this would challenge the neural basis of broadband power in the lower frequency band.
Does low-frequency oscillatory synchronisation (e.g., delta and theta) coordinate MD network communication?	Use electrophysiological functional connectivity analysis and phase-amplitude coupling to measure synchronisation between MD subregions under different cognitive demands. Examine whether task difficulty modulates connectivity patterns between MD nodes.	If low-frequency oscillatory synchronisation mediates MD network communication, increased task demand should enhance synchronisation between MD subregions. Otherwise, it would challenge the role of oscillatory coordination in MD network function.

Beyond these specific experimental tests, there are several broader potential directions for future research that could further refine our understanding of how aperiodic and periodic neural activity contribute to MD network function.

From the perspective of theoretical advancement, first, despite some initial evidence (Kaminski et al., 2017), our understanding of how task demand influences spiking activity in different types of neurons within MD regions remains incomplete. This knowledge is critical for interpreting the observed decrease in aperiodic broadband power alongside increased BOLD signals with demand. Future studies could take advantages of intracranial electrophysiology in humans and non-human primates to explore spiking activity patterns under diverse task demands, bridging neural dynamics across spikes, LFPs, MEG/EEG, and BOLD signals. Second, recent studies suggest that traditional metrics of oscillations based on averaged power may, in fact, be driven by transient oscillatory bursts (Kavanaugh et al., 2024; Lundqvist et al., 2016, 2018). Future research could investigate how oscillatory bursts in different frequency bands contribute to task demand encoding and inter-regional communication within the MD network. This approach would provide a dynamic perspective that contrasts with findings based on averaged power. Third, there is very limited evidence about the role of non-oscillatory FC in cognitive control. Using advanced methods such as wSMI, future studies could examine FC between MD regions and elucidate the unique contributions of non-oscillatory FC to large-scale network-level interactions. Also, investigating the temporal dynamics of aperiodic neural activity in supporting cognitive control remains an important, yet underexplored, area. Taking advantage of newly developed methods (Frelih et al., 2024; Wilson et al., 2022) could offer deeper insights into the real-time roles of aperiodic components in task-related brain activity. In addition, it is a promising direction to develop neural modulation protocols using TMS or transcranial electrical stimulation and to selectively manipulate periodic or aperiodic activity. These techniques could provide causal evidence for the contribution of oscillatory and aperiodic components to MD network function and domain-general cognitive control. Finally, while oscillatory and aperiodic activity have typically been studied in isolation, investigating how these components (as well as their FC patterns) might interact and collaborate to implementing cognitive processes could reveal important insights into the mechanisms underlying cognitive control and the functional architecture of the MD network.

From a practical standpoint, understanding the electrophysiological underpinnings of the MD system has significant implications for cognitive assessment, clinical interventions, and neurotechnology. First, aperiodic broadband power and low-frequency oscillatory synchronisation within the MD network may serve as biomarkers for cognitive function, contributing to the development of MEG/EEG-based cognitive monitoring tools. Additionally, changes in aperiodic brain features have been linked to various psychiatric and neurological conditions, including ADHD, schizophrenia, and age-related cognitive decline (Molina et al., 2020; Robertson et al., 2019; Voytek et al., 2015). Given that MD network activity is critical for adaptive cognitive function, future research could explore whether interventions targeting aperiodic and oscillatory activity in MD regions, using non-invasive brain stimulation, can modulate cognitive performance in these populations. Moreover, insights from MD-related electrophysiology could contribute to artificial intelligence by informing architectures for adaptive, domain-general processing in artificial general intelligence. By studying how the human brain flexibly allocates cognitive resources via aperiodic and oscillatory mechanisms, future research may provide biologically inspired strategies for improving artificial intelligence's ability to generalise across tasks and dynamically adjust processing demands. Together, these applications highlight the broader relevance of electrophysiological research on the MD system, extending its impact beyond cognitive neuroscience into clinical and technological domains.

## Data Availability

No data was used for the research described in the article.
